# Ubiquitin-Specific Peptidase 46 (*Usp46*) Regulates Mouse Immobile Behavior in the Tail Suspension Test through the GABAergic System

**DOI:** 10.1371/journal.pone.0039084

**Published:** 2012-06-14

**Authors:** Saki Imai, Takayoshi Mamiya, Akira Tsukada, Yasuyuki Sakai, Akihiro Mouri, Toshitaka Nabeshima, Shizufumi Ebihara

**Affiliations:** 1 Division of Biomodeling, Graduate School of Bioagricultural Sciences, Nagoya University, Nagoya, Japan; 2 Department of Chemical Pharmacology, Graduate School of Pharmaceutical Sciences, Meijo University, Nagoya, Japan; 3 Division of Applied Genetics and Physiology, Graduate School of Bioagricultural Sciences, Nagoya University, Nagoya, Japan; Université Pierre et Marie Curie, France

## Abstract

The tail suspension test (TST) is widely recognized as a useful experimental paradigm for assessing antidepressant activity and depression-like behavior. We have previously identified ubiquitin-specific peptidase 46 (*Usp46*) as a quantitative trait gene responsible for decreasing immobility time in the TST in mice. This *Usp46* mutation has a 3-bp deletion coding for lysine in the open reading frame, and we indicated that *Usp46* is implicated in the regulation of the GABAergic system. However, it is not known precisely how the immobile behavior is regulated by the GABAergic system. Therefore, in the present study, we examined whether the immobility time is influenced by drugs affecting the action mediated by GABA_A_ receptor using both 3-bp deleted (the *Usp46* mutant) and null *Usp4*6 (*Usp46* KO) mice. Nitrazepam, an agonist at the benzodiazepine-binding site of the GABA_A_ receptor, which potentiates the action of GABA, produced a dose-dependent increase in TST immobility time in the *Usp46* mutant mice without affecting general behaviors. The *Usp46* KO mice exhibited short immobility times comparable to the *Usp46* mutant mice, which was also increased by nitrazepam administration. The effects of nitrazepam in the *Usp46* mutant and KO mice were antagonized by flumazenil. These results indicate that the 3-bp deleted *Usp46* mutation causes a loss-of-function phenotype, and that the GABA_A_ receptor might participate in the regulation of TST immobility time.

## Introduction

The tail suspension test (TST) is widely used for assessing antidepressant activity and depression-like behavior. In this test, mice are subjected to the short-term, inescapable stress of being suspended by their tails. Under such a condition, mice rapidly adopt a characteristic immobile posture that has been named “behavioral despair” on the assumption that the mice have given up hope of escaping. Because antidepressant treatments decrease the immobility time, this test is frequently used for screening drugs for antidepressant activity [1], [2], [3], [4].

The CS mouse is an inbred strain originally established by crossing the NBC and SII strains (both now extinct) at Nagoya University in Japan. CS mice exhibit several distinct phenotypes of circadian behavioral rhythms and sleep properties. For example, CS mice show long free-running periods of over 24 hr (most inbred strains of mice exhibit free-running periods shorter than 24 hrs), spontaneous rhythm splitting and entrainment of circadian rhythms in response to a daily-restricted feeding schedule under constant darkness conditions [5], [6], [7], [8]. In general, abnormal rhythms and sleep patterns are believed to be associated with mental illness. Therefore, we characterized several behavioral phenotypes of CS mice and found that their immobility time in both TST and FST is extremely low (almost no immobility). To identify the gene responsible for this phenotype, we first performed quantitative trait locus (QTL) genetic analysis to map the responsible gene on a chromosome. Subsequently, we produced several congenic or subcongenic strains to narrow the QTL interval, and focused on a candidate gene. To determine the causative gene, we finally rescued the phenotype using bacterial artificial chromosome transgenic mice. Consequently, we identified *Usp46* encoding ubiquitin-specific peptidase as one of the genes responsible for the short immobility time [9].

The *Usp46* mutation that we identified has a 3-bp deletion coding for lysine in the open reading frame. This mutation shortened the duration of loss of righting reflex caused by muscimol (GABA_A_ receptor agonist) administration, and reduced the muscimol-induced GABA_A_ current in hippocampal CA1 pyramidal neurons. In addition, hippocampal expression of the 67-kDa isoform of glutamic acid decarboxylase is decreased in mice with the *Usp46* mutation [9]. Thus, this mutation appears to be implicated in the regulation of the GABAergic system. However, it is not yet known clear exactly how the immobile behavior is regulated by the GABAergic system. In addition, it is not clear whether this 3-bp deletion in *Usp46* is a loss-of-function or gain-of-function mutation. Therefore, in the present study, we addressed these issues using the 3-bp deleted (designated as the *Usp46* mutant mice) and the null *Usp4*6 mice (the *Usp46* KO mice).

## Materials and Methods

### Animals

We purchased C57BL/6J (B6) mice from CLEA Japan Inc. The animals were housed under a 12 hr light/dark cycle (LD 12:12, 7:00 on, 19:00 off) with free access to food and water in our animal facility, at a temperature maintained at approximately 24°C. Only male mice aged between 8 and12 weeks were used. For all experiments, the animals were treated in accordance with the guidelines issued by Nagoya University and Faculty of Pharmaceutical Sciences of Meijo University.

### *Usp46* Mutant Mice

The *Usp46* mutant mice were developed as congenic strains on a B6 genetic background using a marker-assisted breeding strategy. These mice (B6.CS-Ngu1053) contained chromosome 5 regions harboring the *Usp46* of the CS mice [9].

### *Usp46* KO Mice

**Figure 1:**
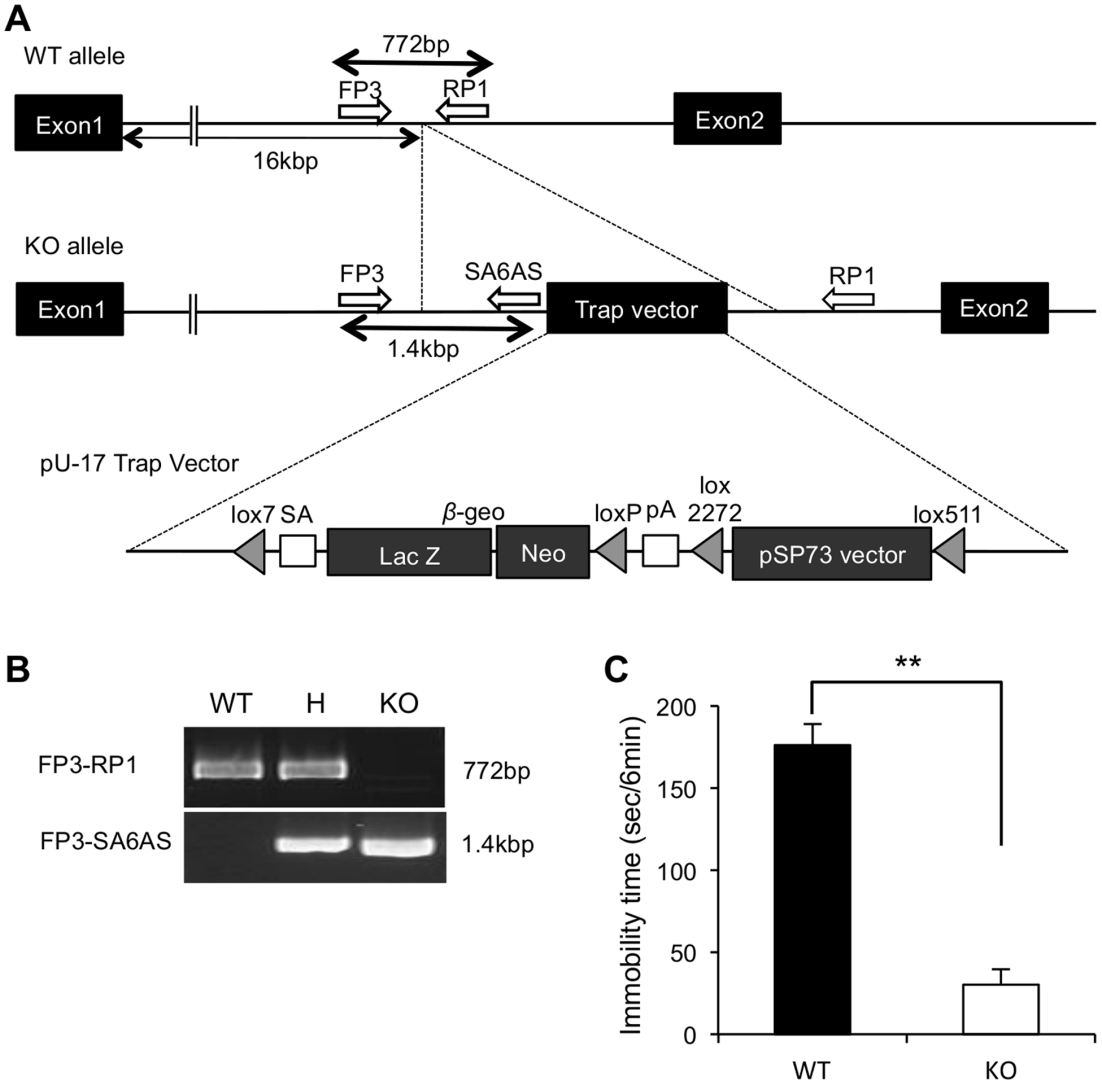
Generation of the *Usp46* KO mice. (A) Integration site of the pU-17 trap vector. The trap vector is inserted approximately 16 kbp downstream from exon 1. The trap vector contains a splice acceptor (SA), the β-galactosidase/neomycin-resistance fusion (β-geo) gene, a polyadeylation signal (pA) and pSP73 vector sequences [10]. The white arrows (FP3, RP1, and SA6AS) indicate the primers used for genotyping. (B) Genotyping by the polymerase chain reaction. DNA fragments of 772 bp from the wild type allele and 1.4 kbp from the inserted allele were amplified by the primer pairs FP3-RP1 and FP3-SA6AS, respectively. (C) Immobility time in wild-type (WT) and *Usp46* KO mice (KO) in the tail suspension test (TST). The KO mice showed a significantly shorter immobility time than the WT mice in the TST. Data represent the mean + S.E.M. for 5–6 mice in each group. **P<0.01 (Student’s *t-*test).

The *Usp46* knockout (KO) mice (17-13906 *Usp46* gene trapped mice, TG Resource Bank #6072) were descendants of the mouse strain generated by Trans Genic Inc. (Kumamoto, Japan) using the gene trap technique (Fig. 1) [10]. Because the original *Usp46* KO mice were generated from B6 and CBA mice, these mice were backcrossed to B6 a minimum of 9 times in order to remove any possible phenotypic variations caused by a different genomic background. To determine their genotypes, ear biopsies were performed at 4 weeks of age for DNA detection using PCR. F1 heterozygous and B6 alleles were amplified using a neo primer pair: 5′-CTGAATGAACTGCAGGACGAG-3′ and 5′-GTCCAGATCATCCTGATCGAC-3′, and lox-SA primer pair: 5′-AGGTCGAGGGACCTATACCG-3′ and 5′-GAGGCCGCTTGTCCTCTTTG-3′. The F1 heterozygous mice were crossed between themselves to obtain the F2 generation: wild-type, heterozygous, and homozygous. To determine their genotypes, FP3 primer 5′-ATAGACTCTGCTGTTTCTCCTATGCTCC-3′, RP1 primer 5′-AATGTTGAGGCAAAGCTGCCAAGCTCAC-3′, and SA6AS primer 5′-CCGGCTAAAACTTGAGACCT-3′ were used.

### Drugs

Nitrazepam (Wako) and flumazenil (SIGMA) were suspended in saline with 0.3% carboxymethylcellulose sodium (SIGMA). Nitrazepam (0.1, 0.3, and 1 mg/kg) was administered intraperitoneally at a volume of 0.1 mL/10 g body weight 30 min before the open-field test (OFT) was conducted. Flumazenil (10 mg/kg) was administered subcutaneously 5 min prior to nitrazepam treatment. Control animals received the same volume of vehicle (saline in 0.3% carboxymethylcellulose).

### Open Field Test (OFT)

Before the behavioral test, mice were moved to the animal room adjacent to the test room, where their behaviors were assayed. The mice were kept there for 1 week under the same conditions as those before being moved. We assayed mice during the light phase (11:00–16:00) after at least a 2 hr adaptation to the test room. Each mouse was placed in the center of a gray plastic box (40×40×40 cm) with the floor divided into 64 compartments (5×5 cm each), and was allowed to freely explore for 5 min under 40 to 50-lx fluorescent light. During the test, the number of grooming, rearing, and climbing (standing on hind legs with forefeet on the wall) behaviors was scored. Because very few instances of rearing were observed, rearing and climbing were combined. After the OFT, the total distance of moving within the box was calculated using SMART software (version 2.0, Panlab, Spain). At the end of the test, the mouse was returned to its home cage, and all apparatus was cleaned with 70% ethanol.

### Tail Suspension Test (TST)

Immediately after the OFT, TST was performed. The procedure for TST was the same as that used in our previous study [9]. Briefly, mice were suspended by their tails using an elastic band attached to the tails by adhesive tape, and the elastic band was hooked onto a horizontal rod. The distance between the tip of the nose of the mouse and the floor was approximately 20 cm. The mice were suspended for a period of 7 min, and the time spent immobile during the last 6 min of the 7 min was recorded for each individual, by an observer blinded to the genotype.

### Northern Blot Analysis

**Figure 2:**
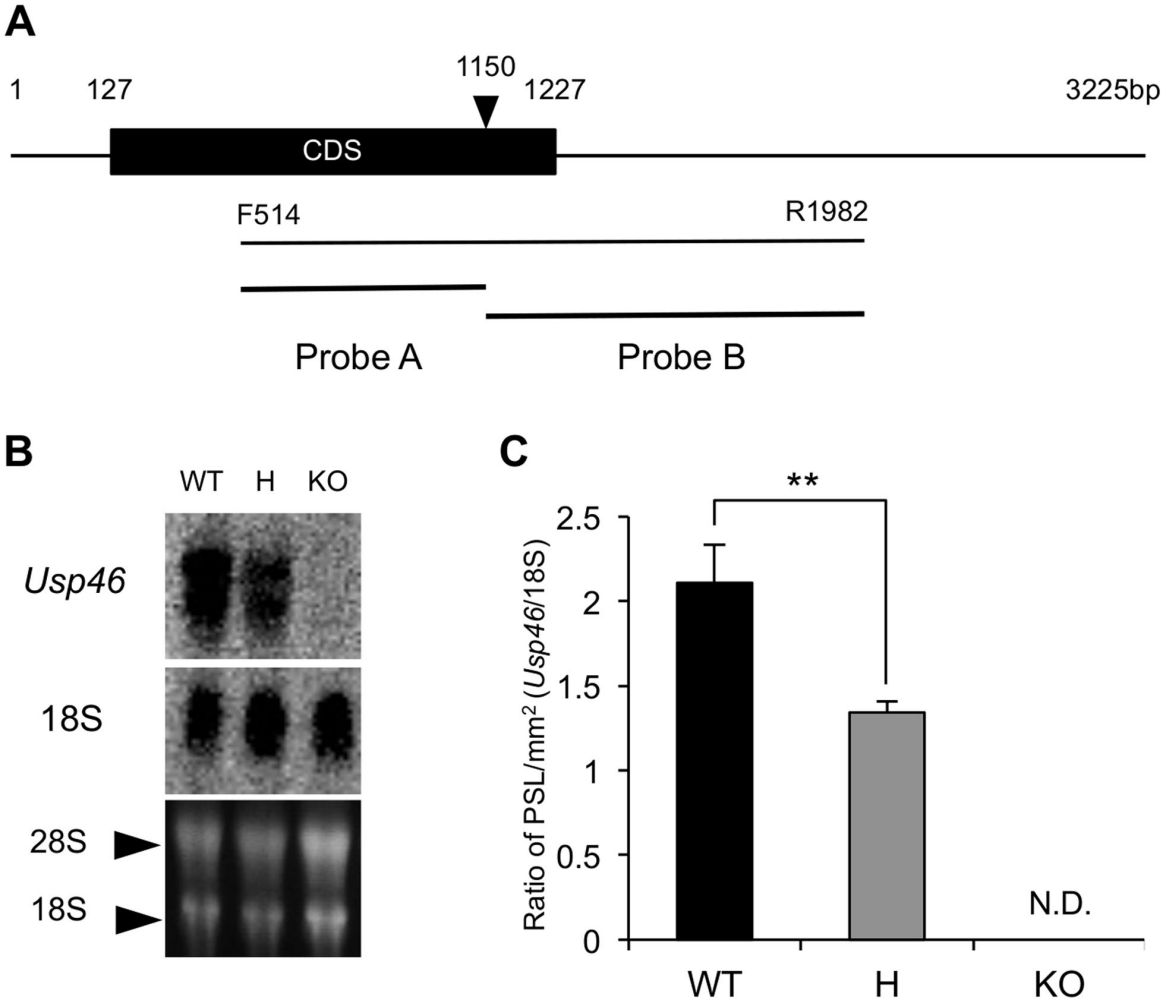
Expression of *Usp46* mRNA in the brain analyzed by northern blot analysis. (A) Schematic diagram of cDNA and probes for *Usp46*. Probes A and B were used for the northern blot analysis. Black arrowhead indicates the *EcoR1* site. F514 and R1982 are a primer pair for PCR. (B) Northern blot from brain total RNA hybridized with the mouse *Usp46* cDNA probes A and B, labeled with [α-^32^P] dCTP. The blot was hybridized with a cDNA probe for S18 as an internal control (the middle panel). The bottom panel shows a photograph of an RNA gel, indicating that equivalent amounts of total RNA were used in each lane. (C) Relative values of *Usp46* mRNA. PSL (Photo stimulated luminescence) was measured. Signals from the *Usp46* KO mice (KO) were not detected (N.D.). The differences between the wild-type mice (WT) and the heterozygotic mice (H) were significant (**P<0.01, n = 6, Student’s *t*-test).

Male mice were killed by decapitation and the brains were collected, frozen in liquid nitrogen, and stored at −80°C until RNA extraction was conducted. Total RNA was extracted using TRIZol Reagent (Invitrogen, Carlsbad, USA) and quantified by absorbance at 260 nm (1 optical density unit = 40 µg RNA/mL). Total RNA (1 µg) was reverse transcribed into complementary DNA (cDNA) using the Moloney murine leukemia virus (M-MLV) reverse transcriptase and oligo(dT) primer. The cDNA was amplified by 45 cycles of PCR using mouse *Usp46* specific primers (F514: 5′-AACACTATTGCGGACATCCTG-3′ and R1982: 5′-AAAGCCACGTTTCTGGAAAAT-3′). The PCR cycle consisted of 10 s of denaturation at 98°C, 30 s of annealing at 60°C, and 2 min of extension at 72°C. The amplified product was cloned into the pGEM-T easy™ plasmid vector and sequenced by the dideoxy chain termination method using a BigDye Terminator v3.1 Cycle Sequencing Kit (Applied Biosystems). The DNA fragments were used as probes for northern blot analyses. The location of these probes on the *Usp46* cDNA is shown in Figure 2A. Total RNA samples were separated on 1% (w/v) agarose gel containing 2.2 M formaldehyde. The separated RNA samples were blotted onto a Hybond+ nylon membrane (GE Healthcare, BKM, UK) and hybridized to the mouse *Usp46* cDNA probe labeled with [α-^32^P] dCTP. After hybridization, the membrane was washed sequentially in 2×SSC containing 0.1% SDS at 62°C for 30 min, 0.1×SSC containing 0.1% SDS at 62°C for 30 min twice. The amount of mRNA was determined by a Fuji BAS 2000 Bioimaging Analyzer (Fujifilm, Tokyo, Japan).

### Statistics

The data are expressed as mean + S.E.M. Significant differences between the 2 groups were determined using Student’s *t*-tests. One-way ANOVA with the Tukey-Kramer test, or two-way ANOVA with Bonferroni’s test were used for multiple comparisons.

## Results

### *Usp46* KO Mice

The trap vector was inserted into an intron of *Usp46* approximately 16 kbp downstream from exon 1 (Fig. 1A). A PCR product of 772 bp was detected in the wild-type mice, but not in the KO mice. Instead, 1.4 kbp products were detected in the KO mice. In the heterozygous mice, as expected, both products were observed (Fig. 1B). To confirm the absence of *Usp46* mRNA expression in the brains of the KO mice, we performed northern blot analysis. The results showed the absence, and approximately half the level of *Usp46* mRNA in the brains of KO mice and heterozygous mice, respectively (Fig. 2B, C). We also confirmed that the KO mice showed significantly shorter immobility times than the wild-type mice in TST (Fig. 1C).

### Dose-dependent Effects of Nitrazepam on TST Immobility Time in *Usp46* Mutant Mice

**Figure 3:**
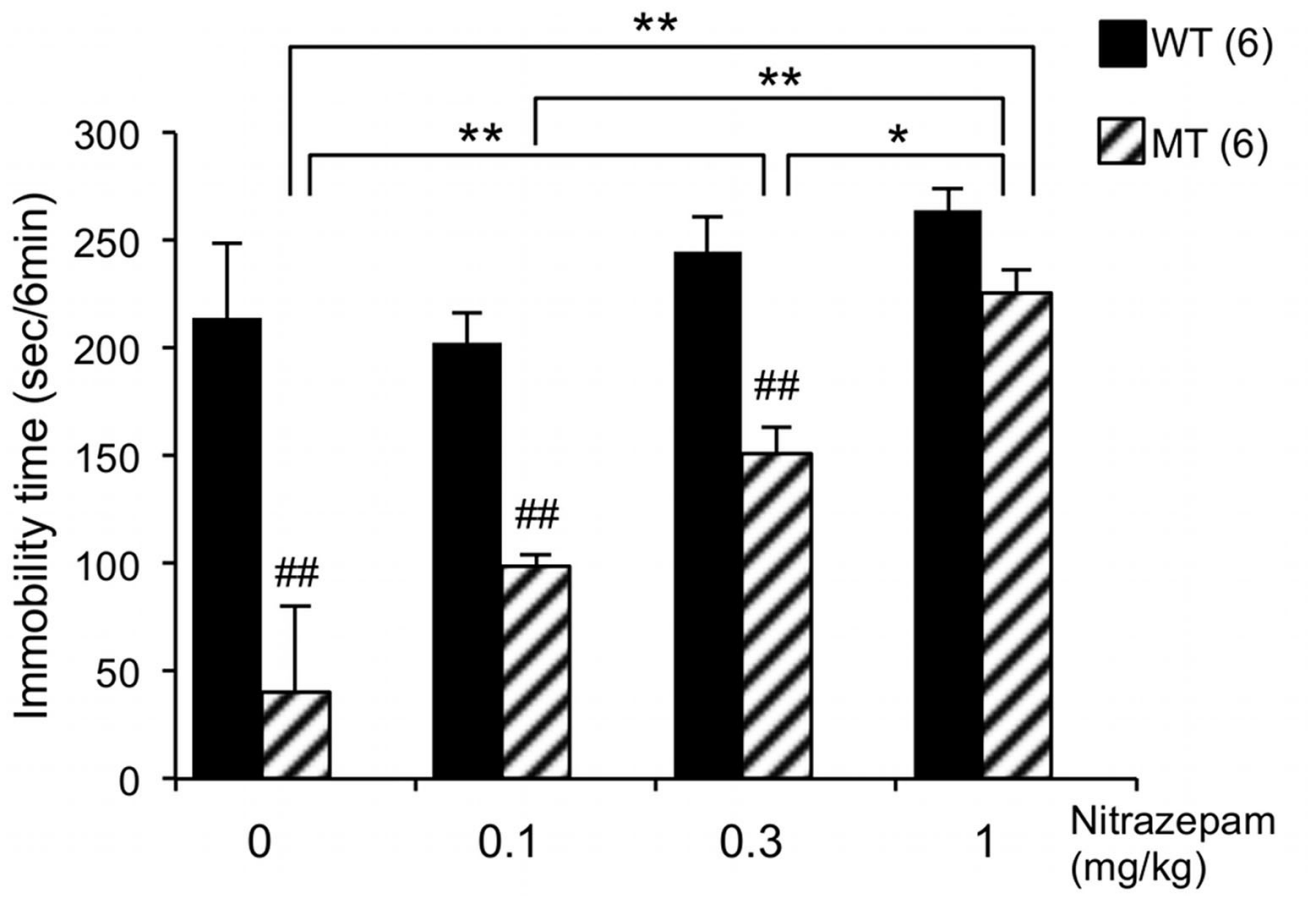
Effects of nitrazepam administration on tail suspension test (TST) immobility time. Nitrazepam induces dose-dependent increases of TST immobility time in *Usp46* mutant mice (MT). Data are expressed as mean + S.E.M. for 6 mice in each group. ^##^ *P*<0.01 compared with the wild-type mice for each dose; *P<0.05, **P<0.01 (two-way ANOVA with Bonferroni’s test).

Nitrazepam, an agonist of the benzodiazepine-binding site of GABA_A_ receptors that causes an enhanced binding of GABA to these receptors, produced a dose-dependent increase in TST immobility times in the *Usp46* mutant mice (*F*_dose (3,40)_ = 19.09, *P*<0.01; *F*_genotype(1,40)_ = 73.56, *P*<0.01; *F*_dose×genotype(3,40)_ = 5.36, *P*<0.01; two-way ANOVA) (Fig. 3). However, such a dose-dependent increase was not apparent in the wild-type mice, although a significant difference between doses of 0.1 mg/kg and 1 mg/kg was observed. Because nitrazepam is known to have sedative effects, we measured general behaviors in OFT. However, an administration of this drug did not affect general behaviors (total activity, frequency of climbing + rearing, and grooming for 5 min in OFT) (*P*>0.05; one-way ANOVA with Tukey-Kramer post-hoc test) (Table 1), while nitrazepam affected TST immobility times in the *Usp46* mutant mice.

**Table 1:** Effects of nitrazepam (1 mg/kg) and flumazenil (10 mg/kg) on general behaviors in OFT.

Behavior	Genotype	Saline	Nitrazepam	Nitrazepam +Flumazenil
TotalActivity (cm)	WT (6)	1489.9±191.4	1807.7±151.2	1296.7±83.8^**^
	MT (6)	2142.5±115.3	1840.8±173.4	1670.6±433.6
	KO (5)	2417.6±223.3	1763.8±194.4	2421.1±440.0
Rearing +Climbing	WT (6)	17.3±5.6	32.5±3.4	19.0±2.2
	MT (6)	29.3±4.2	21.3±3.9	24.6±6.6
	KO (5)	33.0±5.6	29.0±5.5	39.6±6.3^*^
Grooming	WT (6)	1.2±0.3	1.0±0.4	2.6±0.9
	MT (6)	1.0±0.3	0.8±0.2	2.0±0.6
	KO (5)	1.2±0.5	1.2±0.3	2.8±0.7

Data are shown as the mean ± S.E.M. The number of animals is given in parentheses. **P<0.01 compared with the saline-treated group in wild-type (WT) mice, *P<0.05 compared with the saline-treated group in the *Usp46* KO mice (KO) (Tukey-Kramer post-hoc test).

### Effects of co-administration of Nitrazepam and Flumazenil on TST Immobility Time in *Usp46* Mutant and KO Mice

As seen in Fig. 3, nitrazepam (1 mg/kg) completely restored the immobility times to a level of wild-type in the *Usp46* mutant mice. Therefore, we used this dose to ascertain whether the restored immobility time is blocked by flumazenil, an antagonist at the benzodiazepine-binding site of the GABA_A_ receptor [11]. Prior to this test, we examined the effects of a single administration of flumazenil on TST immobility time and general behaviors (total activity, frequency of grooming, and climbing + rearing for 5 min in OFT) in both the *Usp46* mutant (n = 6) and the wild-type mice (n = 6) (all mice used were naive), and found that there were no significant effects of flumazenil on these behaviors in both types of mice (data not shown).

**Figure 4:**
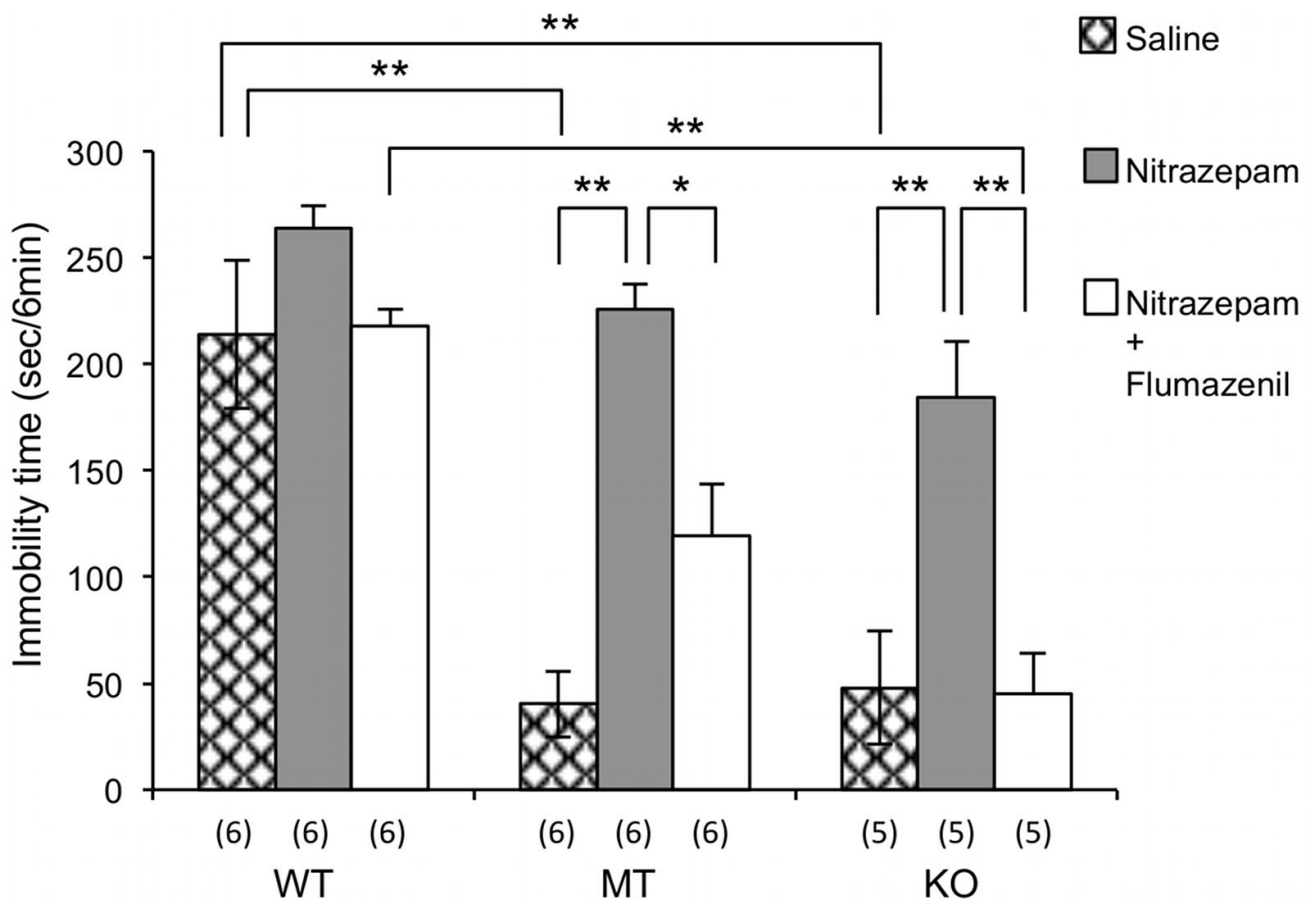
Effects of administration of nitrazepam and flumazenil on tail suspension test (TST) immobility time. Nitrazepam (1 mg/kg) causes a significant increase in immobility time in the TST, which was blocked by flumazenil (10 mg/kg) in both the *Usp46* mutant (MT) and in the *Usp46* KO mice (KO). In the wild-type mice (WT), no significant effects were observed with either single administration of nitrazepam, or co-administration of nitrazepam and flumazenil. Data are expressed as mean + S.E.M. for 5–6 mice in each group. *P<0.05, **P<0.01 (two-way ANOVA with Bonferroni’s test).

To investigate whether the GABA_A_ receptor is involved in the regulation of immobility time in TST, the antagonizing effects of flumazenil on the nitrazepam-induced increase of immobility time were examined. As a result, we observed that the enhanced TST immobility time elicited by nitrazepam was blocked by flumazenil in both *Usp46* mutant and KO mice (*F*_treatment (2,42)_ = 28.52, *P*<0.01; *F*_genotype(2,42)_ = 35.35, *P*<0.01; *F*_treatment×genotype(2,42)_ = 3.47, *P*<0.05; two-way ANOVA with Bonferroni’s test; Fig. 4). Co-administration of these drugs had mild effects on total activity levels in wild-type mice, and on the frequency of rearing + climbing behavior in KO mice (Table 1).

## Discussion

In the present study, we addressed 2 primary questions: (1) whether *Usp4*6 KO mice show a similar phenotype to *Usp46* (3-bp deleted) mutant mice in TST; and (2), whether the immobility time is regulated by the GABAergic system.

The KO mice with the insertion of the trap vector, which contains the β-galactosidase gene in the region between exon 1 and exon 2, did not express *Usp46* within the brain. As shown in Fig. 1, *Usp46* KO mice showed significantly shorter immobility time than wild-type mice. This phenotype in *Usp46* KO mice was similar to *Usp46* mutant mice (Fig. 3, 4) [9]. Additionally, we observed β-galactosidase expression by immunohistochemistry in the hippocampus, one of the brain regions that strongly expresses *Usp46*, in the KO mice (unpublished results). These results indicate that the 3-bp coding for lysine is important for USP46 deubiquitinating enzyme to be functionally active, and suggests that this mutation causes a loss-of-function phenotype. Indeed, recently, it has been reported that this 3-bp deletion decreases USP46 deubiquitinating enzyme activity [12].

Previously, we have reported that *Usp46* is implicated in the regulation of the neuronal GABAergic system, based on results showing that the sensitivity to muscimol (a selective GABA_A_ receptor agonist and a partial agonist for GABA_C_ receptors) is attenuated in the muscimol-induced righting reflex and the muscimol-induced inhibitory current in CA1 pyramidal neurons of the hippocampus of *Usp46* mutant mice [9]. However, there is no direct evidence that attenuation of the GABAergic system alters immobile behavior in the TST. In this study, therefore, we addressed this issue and found that the TST immobility time was increased by nitrazepam in a dose-dependent manner in *Usp46* mutant mice, as compared to the wild-type mice, in which the immobility time slightly increased only at the highest dose (Fig. 3). This attenuation was blocked by flumazenil in both the *Usp46* mutant and in the KO mice (Fig. 4). Additionally, nitrazepam did not significantly change total activity levels, frequency of grooming, or climbing + rearing behaviors in the OFT (Table 1). Furthermore, it is known that a low dose of nitrazepam, which has no sedative effects, does not induce sleep in mice [13]. Additionally, there are some reports that the GABAergic system might be involved in the regulation of TST immobility time; the GABA_A_ receptor α3 subunit was identified as a candidate gene affecting immobility time in the TST using QTL analysis [14], Gabra3 KO mice have exhibited low immobility times in FST [15], GABA_A_ receptor α2 subunit KO mice have showed longer immobility times in the TST [16], and GABA transporter subtype 1 (GAT1) KO mice have showed shorter immobility times in both the TST and FST [17]. Further, it is reported that GAD65 (a 65-kDa isoform of glutamate decarboxylase) KO mice exhibit decreased immobility times in the FST [18]. In addition, genetic analysis for immobility times in both TST and FST identified significant QTL on the chromosomal region that harbors the GABA_A_ receptor subunit [19], [20]. Taken together, these results suggest that the decrease of immobility times in the TST as shown by our mice is regulated by an attenuation of the action of the GABAergic system produced by the *Usp46* mutation.

It is not known how *Usp46*, which encodes a deubiquitinating enzyme, affects the GABAergic system. Recently, it has been reported that USP46 regulates the abundance of the glutamate receptor GLR-1 in the ventral nerve cord of *Caenorhabditis elegans*. Because of the relatively high homology in amino acid sequences for USP46 between the mouse and *C. elegans* (60% identical), it is interesting to contemplate whether USP46 function similarly in mammalian species [21].

In summary, we have found that *Usp46* KO mice show low TST immobility time which is similar to *Usp46* mutant mice, and this immobile behavior is regulated by the GABAergic system. These *Usp46* mutant and KO mice may be useful for understanding other impaired behaviors which are attributed to GABAergic alterations induced by *Usp46* dysfunction in mice.
